# Redefining Peptide 14D: Substitutional Analysis for Accelerated TB Diagnosis and Enhanced Activity against *Mycobacterium tuberculosis*

**DOI:** 10.3390/microorganisms12010177

**Published:** 2024-01-16

**Authors:** Kai Hilpert, Tulika Munshi, Paula M. López-Pérez, Joana Sequeira-Garcia, Tim J. Bull

**Affiliations:** 1 Institute of Infection and Immunity, St George’s, University of London, Cranmer Terrace, London SW17 0RE, UK; 2 TiKa Diagnostics Ltd., Cranmer Terrace, London SW17 0RE, UK

**Keywords:** antimicrobial peptide, *Mycobacterium tuberculosis*, diagnosis, Spot synthesis, substitution analysis, TB, anti TB compound

## Abstract

Tuberculosis (TB) caused by *Mycobacterium tuberculosis* remains a predominant cause of mortality, especially in low- and middle-income nations. Recently, antimicrobial peptides have been discovered that at low concentrations could stimulate the growth of *M. tuberculosis* (hormetic response). In this study, such a peptide was used to investigate the effects on the time to positivity (TTP). A systematic substitution analysis of peptide 14D was synthesized using Spot synthesis technology, resulting in 171 novel peptides. Our findings revealed a spectrum of interactions, with some peptides accelerating *M. tuberculosis* growth, potentially aiding in faster diagnostics, while others exhibited inhibitory effects. Notably, peptide NH_2_-wkivfiwrr-CONH_2_ significantly reduced the TTP by 25 h compared to the wild-type peptide 14D, highlighting its potential in improving TB diagnostics by culture. Several peptides demonstrated potent antimycobacterial activity, with a minimum inhibitory concentration (MIC) of 20 µg/mL against H37Rv and a multidrug-resistant *M. tuberculosis* strain. Additionally, for two peptides, a strongly diminished formation of cord-like structures was observed, which is indicative of reduced virulence and transmission potential. This study underscores the multifaceted roles of antimicrobial peptides in TB management, from enhancing diagnostic efficiency to offering therapeutic avenues against *M. tuberculosis*.

## 1. Introduction

Tuberculosis (TB) is a severe infectious disease that primarily affects the lungs but can also impact other parts of the body, caused by the bacterium *Mycobacterium tuberculosis* (MTB). TB is one of the top 15 causes of death worldwide, with a mortality of 1.3 million people in 2022 [1]. The disease has a significant presence in low- and middle-income countries, over 80% of cases and deaths occur there, due to socio-economic factors such as poverty, malnutrition, and inadequate healthcare systems [2,3,4,5,6,7,8,9].

Culture diagnostics of TB have long been considered the gold standard for TB diagnosis due to their high sensitivity and specificity [10]. They allow for the direct isolation and identification of the *Mycobacterium tuberculosis* complex from clinical specimens, providing a definitive diagnosis. Moreover, culture diagnostics enable drug susceptibility testing, which is crucial for identifying drug-resistant strains and tailoring treatment regimens [11]. Molecular and immunological diagnostics serve as complementary tools, offering rapid preliminary results, while culture diagnostics provide confirmatory testing and drug susceptibility profiles [10].

The cord factor Trehalose 6, 6′-dimycolate (TDM), is the most abundant glycolipid in the mycobacterial cell wall of *Mycobacterium tuberculosis*. Similar to a biofilm formation of other bacteria, the cord provides a dense arrangement of bacteria that leads to a shield against antibiotic-mediated killing. It displays well-characterized conformation-dependent immunostimulatory effects, that enable the bacteria to suppress host inflammatory response when needed. The cords play a role in bacterial dissemination by exerting forces on cellular organelles and tissues. This might aid in the spread of the infection within the host, making it more challenging to contain and treat [12,13].

Antimicrobial peptides (AMPs) are a class of small, naturally occurring peptides known for their broad-spectrum antimicrobial activity. They are ubiquitous in nature, found in almost all living organisms, and in higher organisms play a crucial role in innate immunity [14,15,16,17,18,19,20,21,22,23]. AMPs show a very strong sequence diversity, with more than 20,000 unique peptides known [24]. There is also a structural diversity of AMPs, including α-helical, β-sheet, extended structures, and structure combinations [25]. Most cationic AMPs demonstrate a strong affinity for binding with negatively charged lipopolysaccharides (LPSs) in Gram-negative bacteria, as well as with similarly negatively charged lipoteichoic acids in Gram-positive bacteria. Following this binding, they interact with and often cause depolarization and/or increased permeability in the cytoplasmic membranes [26]. In numerous instances, the peptide moves into the microbial cell to target internal structures. Hence, AMPs have the potential to impact a variety of processes as they typically do not have a single target, but multiple [27,28,29,30,31,32,33,34,35,36,37,38]. It is documented that certain AMPs have an affinity for lipid II, DNA and RNA, chaperones and ribosomes, proteases, adenosine triphosphate (ATP), and ABC transporters [39,40,41,42,43,44,45,46]. At sub-lethal concentrations, certain antimicrobial peptides (AMPs) and antibiotics are known to enhance growth, motility, mutation frequency, and plasmid conjugative transfer [47]. An example of this is the GL13K peptide, which has been observed to stimulate growth and increase metabolic activity in *Pseudomonas aeruginosa* [48]. This effect is commonly described as hormesis. Our research has identified peptides exhibiting hormetic responses in slow-growing mycobacteria, including *Mycobacterium tuberculosis* (MTB) [49]. One particular peptide, peptide 14D (NH_2_-wkivfwwrr-CONH_2_) has already been shown to improve diagnostics of *Mycobacterium avium* subspecies *paratuberculosis* (MAP) and *Mycobacterium bovis* in various animal samples including wildlife samples from the Kruger National Park in South Africa [50,51,52].

In a recent clinical trial involving 255 samples from adults, peptide 14D, combined with another peptide, significantly reduced the time to positivity (TTP) for MTB detection. This combination resulted in a 46% increase in sample positivity rates using a routine test [53]. In addition, the interaction of 14D with membranes was measured, as well as the gene expression response for *M. tuberculosis* and *M. bovis* [53]. In addition, the interaction of 14D with membranes was measured as well as the gen expression response was determined for *M. tuberculosis* and *M. bovis* [53]. Consequently, our current focus is on investigating the potential of peptide 14D to further decrease TTP in commercial assays. We have conducted a substitution analysis on peptide 14D, generating 171 new variants. Those substitutions provided information about what makes the peptides more active or drives it towards inhibition instead of stimulation. The TTP for all variants was determined, and selected variants were re-synthesized on resin and purified to confirm the screening results.

## 2. Methods

### 2.1. Peptides

The peptide library was fabricated utilizing automated solid-phase peptide synthesis (SPPS) on a Whatman 50 cellulose membrane (10 cm × 15 cm) with the assistance of a MultiPep RSI peptide synthesizer (Intavis, Tuebingen, Germany), adapting the manual synthesis protocol delineated in [54]. Initially, the membrane underwent functionalization through an overnight incubation in a solution comprising 0.2 M Fmoc-Gly-OH (Aldrich, Gillingham, UK), 0.24 M N,N′-diisopropyl carbodiimide (DIC, Fluka, Loughborough, UK), and 0.4 M N-methylimidazole (NMI, Aldrich) in dimethylformamide (DMF, VWR, Leicestershire, UK), setting the stage for automated synthesis.

Following this, deprotection of the glycine was carried out in a 20% piperidine solution (*v*/*v*, Thermofisher Acros Organics, Geel, Belgium) in DMF, executed in two phases of 20 and 10 min. The synthesis at distinct spots utilized the Fmoc/tBu strategy, employing pre-activated Fmoc amino acids (Bachem, Bubendorf, Switzerland) mixed with equal molar quantities of 1-hydroxybenzotriazole hydrate (HOBt, Aldrich, Gillingham, UK) and DIC, all housed in N-methyl-2-pyrrolidone (NMP, VWR, Leicestershire, UK). This approach ensured heightened coupling efficiency at each position of the amino acid sequence through a double coupling procedure lasting 2 × 10 min.

Post each amino acid coupling cycle, a 5 min treatment with acetic anhydride (5% *v*/*v* in DMF, Fluka) was administered to cap unreacted residues. Subsequently, the Fmoc protective group was removed using a 20% piperidine solution in DMF for two intervals of 5 min each. The final stage of the synthesis involved the cleavage of the amino acid side-chain-protecting groups, executed through a two-step process involving solutions with varying concentrations of trifluoroacetic acid (TFA) (Acros Organics, Geel, Belgium), tri-isopropylsilane (TIPS, Acros Organics, Geel, Belgium), and water in dichloromethane (DCM, Acros Organics, Geel, Belgium).

The peptides were then severed from the solid support following an overnight incubation in a saturated atmosphere of ammonia gas. The yield and quality of the SPOT synthesis were ascertained using a control peptide and a subset of peptides from the synthesis (*n* = 18). These peptides were isolated using a one-hole-puncher (Ø = 6 mm), placed in a sterile 96-well round-bottomed polypropylene non-treated microtiter plate, and left to dissolve overnight in 200 µL of sterile water at room temperature. The peptide concentration was quantified through absorbance measurements at 280 nm using a spectrophotometer. Further analysis was conducted using analytical RP HPLC on a Shim-pack VP-ODS column (120 Å, 150 × 4.6 mm, Shimadzu, Milton Keynes, UK) facilitated by an LC2010AHT system (Shimadzu, Milton Keynes, UK), employing a solvent system with specific gradients and flow rates.

Peptides on resin were synthesized through automated solid-phase peptide synthesis (SPPS) utilizing a MultiPep RSI peptide synthesizer (Intavis, Tuebingen, Germany) and adhering to the 9-fluorenyl-methoxycarbonyl-tert-butyl (Fmoc/tBu) strategy. The reactive side chains were safeguarded using protective groups such as tBu for Tyr and Asp, trityl (Trt) for Asn, Cys, Gln, and His, 2,2,4,6,7 pentamethyldihydrobenzofuran-5-sulfonyl (Pbf) for Arg, and tert-butoxycarbonyl (Boc) for Lys and Trp.

During the automated SPPS, four equivalents of Fmoc amino acids (Bachem, Bubendorf, Switzerland) were coupled onto TentaGel^®^ HL RAM resin (25 μmol scale, loading 0.3–0.4 mmol/g, Rapp Polymere, Tuebingen, Germany). This was facilitated by in situ activation involving four equivalents of N,N,N′,N′-Tetramethyl-O-(1H-benzotriazol-1-yl)uronium hexafluorophosphate (HBTU; Carbosynth, Berkshire, UK) and eight equivalents of N-Methylmorpholine (NMM, Sigma, Dorset, UK). A double-coupling procedure was executed for 2 × 30 min, followed by the removal of the Fmoc group using a 20% (*v*/*v*) piperidine solution (Thermofisher Acros Organics, Geel, Belgium) in dimethylformamide (DMF, Jencons-VWR, Leicestershire, UK).

The peptide amides were then cleaved from the resin using a 95% (*v*/*v*) aqueous trifluoroacetic acid (TFA, Fisher Scientific, Loughborough, UK) solution, which contained a 5% (*v*/*v*) triisopropylsilane (TIPS, Thermofisher Acros Organics, Geel, Belgium)/water (1:1) scavenger mixture, a process lasting 3 h. Following this, the cleaved peptides were precipitated using ice-cold methyl tert-butyl ether (MTBE; Thermofisher Acros Organics, Geel, Belgium), and post centrifugation, they were dissolved in a solution containing 20% (*v*/*v*) acetonitrile (ACN, Jencons-VWR, Leicestershire, UK) and 80% (*v*/*v*) water with 1% (*v*/*v*) TFA, achieving a concentration of 15 mg/mL.

Analytical reversed-phase (RP) HPLC on a Shim-pack VP-ODS column (120 Å, 150 × 4.6 mm, Shimadzu, Milton Keynes, UK) facilitated by a Shimadzu LC2010AHT system (Shimadzu, Milton Keynes, UK) was employed for analysis. The solvent system used contained 0.1% (*v*/*v*) TFA in H_2_O (solvent A) and 0.1% (*v*/*v*) TFA in acetonitrile (solvent B). Verification of the identity was conducted through liquid chromatography–electrospray ionisation mass spectrometry (LC-ESI-MS) using a Shimadzu LC2020 system (Shimadzu, Milton Keynes, UK) equipped with a Jupiter 4 μ Proteo C18 column (90 Å, 250 × 4.6 mm, Phenomenex, Cheshire, UK). The solvent system here comprised 0.01% (*v*/*v*) TFA in H_2_O (solvent A) and 0.01% (*v*/*v*) TFA in acetonitrile (solvent B).

Crude peptides were purified to the homogeneity of >92% by preparative RP HPLC on a Shimadzu LC2020 system equipped with a Jupiter 10 μ Proteo C18 column (90 Å, 250 × 21.2 mm, Phenomenex, (Phenomenex, Cheshire, UK)) using a linear gradient system containing 0.01% (*v*/*v*) TFA in H_2_O (solvent A) and 0.01% (*v*/*v*) TFA in acetonitrile (solvent B). Pure products were finally characterized by analytical RP-HPLC and LCMS.

### 2.2. Mycobacterial Culture

The *Mycobacterium tuberculosis* laboratory strain H37Rv was cultivated in Middlebrook 7H9 media (BD, Wokingham, UK) supplemented with 0.2% glycerol, 0.1% casitone, and 10% OADC at 37 °C. The screening was automated using the BACTEC MGIT 320 system with MGIT mycobacterial culture tubes, supplemented according to manufacturer recommendations. For the determination of MIC, a multidrug-resistant clinical isolate from St George’s Hospital was used.

Cultures were prepared for microscopy by air drying onto standard microscope slides and then fixed in 10% formalin for 3 h. Light Microscopy was performed by the Image Resource Facility, St Georges University using a Nikon Ni-E microscope fitted with a Nikon DS-Fi3 camera(Nikon-UK, Surbiton, UK). Nikon NIS-Elements software (BR 5.20.02) was used to process the images.

### 2.3. Peptide Screening

Peptide screening and verification assays were conducted using the BACTEC MGIT 320 system, with MGIT mycobacterial culture tubes supplemented as per manufacturer guidelines. The tubes were inoculated with exponential starter cultures and peptides at different final concentrations, and TTPs were taken from the automated reader, measuring the relative fluorescence of an oxygen depletion indicator. Once a preset threshold is crossed, the TTP is given.

### 2.4. Minimal Inhibitory Concentration

The determination of the Minimal Inhibitory Concentration (MIC) was carried out in 96-well plates utilising a microdilution assay, as delineated by Wiegand et al. [55]. In summary, peptide 14D was incorporated into the inoculum through a stepwise dilution series, achieving a final bacterial concentration of 2 × 10^5^ CFU/mL in the assay. The 96-well plates were then sealed with a plate sealer and incubated at 37 °C for 14 days. To assess viable bacteria, resazurin was used, with an incubation period of 48 h at 37 °C. This assay was conducted twice, with each experiment comprising three technical replicates. Given that all six trials yielded consistent results, a third iteration was deemed unnecessary, thereby conserving both time and resources in the biosafety category 3 laboratory.

## 3. Results

### 3.1. Determining the Time to Positivity (TTP) for All 171 Single Substitutions of Peptide 14D

**Figure 1:**
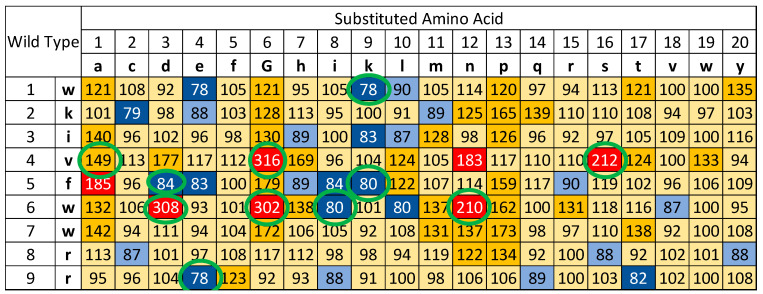
Substitutional analysis of peptide 14D (NH_2_-wkivfwwrr-CONH_2_) regarding the Time to Positivity (TTP) of *Mycobacterium tuberculosis*. All 14D controls (18) were set to 100%, and all other values are relative to this benchmark. The activity levels of the substitutions are indicated by a colour-coding system in our data visualization: dark blue represents values less than 85%, indicating substitutions with the potential for enhanced growth stimulation. Light blue signifies values between 85% and 90%, which indicate substitutions with the potential for marginally increased growth stimulation. Light orange is used for values between 90% and 120%, corresponding to activity levels akin to the wild type. Orange denotes values between 120% and 180%, suggesting substitutions with potential for medium growth inhibition. Finally, red represents values greater than 180%, which denote substitutions with the potential for a strong growth inhibition. Additionally, green circles are used to mark the substitutions selected for verification.

Spot synthesis is a time- and cost-efficient method for the synthesis of large numbers of peptides in a parallel and addressable fashion and was developed by Ronald Frank [56]. This technique is ideal for screening assays since the cost of peptides is low and production time is fast because of the highly parallel synthesis and the fact that peptides are not purified. Reliability and purity of the peptides are reported frequently [57,58,59,60,61,62,63,64]. This technique was employed to synthesise a systematic substitution library of the peptide 14D (NH_2_-wkivfwwrr-CONH_2_), comprising 171 new peptides. These peptides were solubilised in water, and their absorbance at 280 nm was measured to adjust the concentration. The experiment was conducted at a concentration of 3 µg/mL. Each individual peptide was transferred to a separate 7 mL MGIT (Becton Dickinson, UK) mycobacterial culture tube and inoculated with an exponential starter culture. Subsequently, the tube was placed into a BACTEC MGIT 320 automated mycobacterial detection system, and the time-to-positivity (TTP) was monitored. The TTP values for the peptide 14D controls were set at 100%, and all other TTP values were calibrated relative to this benchmark. Consequently, we have delineated different activity groups. Values less than 85% signify substitutions with potential for enhanced growth stimulation; values between 85% and 90% indicate substitutions with potential for marginally increased growth stimulation; values from 90% to 120% are akin to the wild type; values between 120% and 180% suggest substitutions with potential for medium growth inhibition; and values exceeding 180% denote substitutions with a potential for strong growth inhibition. This data is presented in Figure 1 and color-coded for improved visual interpretation.

Of the 171 peptides analysed, 11 exhibited a Time to Positivity (TTP) smaller than 85%, while 13 peptides demonstrated a TTP ranging between 85 and 90% when compared to the wild-type peptide. From the most active set, five peptides were selected for subsequent verification.

Seven peptides displayed pronounced growth inhibition, surpassing 180%. From this subset, five peptides were chosen for further validation. A total of 36 peptides showed a growth inhibition between 120 and 180%, with 1 peptide from this category being selected for additional validation. Notably, a majority of peptides (104) exhibited activity analogous to the wild-type peptide, falling within the 90 to 120% range.

### 3.2. Verification of the Screen with Purified Peptides

As described above, a total of 11 peptides were selected for verification of the results. These peptides were synthesized on resin and purified with HPLC-MS. The results are given in Table 1.

In the initial screening, a concentration of 3 µg/mL of the peptide was utilised. For subsequent verification, concentrations of 0.3, 3, 10, and 30 µg/mL were selected to elucidate dose-dependent effects. Focusing on the 3 µg/mL dosage for comparison and verification, eight (73%) of the eleven purified peptides exhibited similar levels of activity as measured in the initial screen. This observation corroborates the screening data. The three peptides displaying divergent activity were all categorised as “substitutions with potential for enhanced growth stimulation”. One demonstrated only a minor enhancement in growth, while the other two were inhibitory. The most efficacious peptide in terms of growth stimulation, NH_2_-wkivfiwrr-CONH_2_, reduced the Time to Positivity (TTP) by 25 h compared to the wild-type peptide and by 56 h relative to the untreated control. The peptide NH_2_-wkivfdwrr-CONH_2_ exhibited the most pronounced inhibition at 3 µg/mL, prolonging the TTP by approximately 22 days compared to the wild type. Consequently, the majority of the purified peptides demonstrated effects consistent with the screening results, thus validating the outcomes of the initial screen.

### 3.3. Determining the Minimal Inhibitory Concentration (MIC) of Selected Peptides with a Strong Inhibitory Effect

Here, we applied the microdilution broth assay for five peptides. The results of the MICs are shown in Table 2.

In the observed increase in TTP values (see Table 1) associated with these peptides, each exhibited a reduced MIC in comparison to the wild-type peptide 14D. At a concentration of 30 µg/mL, every peptide extended the TTP by approximately 40 days relative to *M. tuberculosis* alone. Notably, the minor variations observed in TTP reduction did not correspond to distinct MICs; all were consistently measured at 20 µg/mL. As is commonly observed with numerous antimicrobial peptides, the MIC against multi-drug resistant (MDR) strains remains consistent with that of the sensitive strains. This pattern is evident in the current data as well.

### 3.4. Observing Changes in Cord Formation in the Presence of Peptides at 10 µg/mL

**Figure 2:**
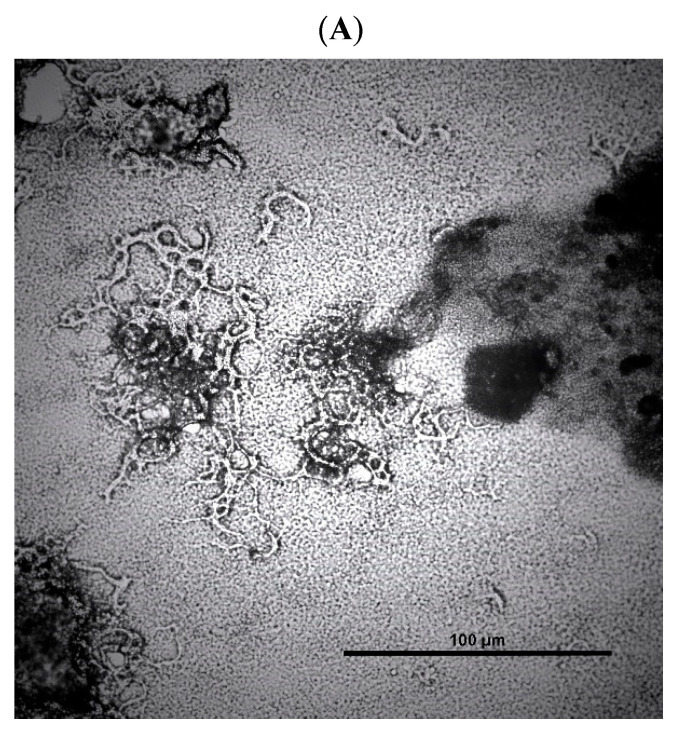

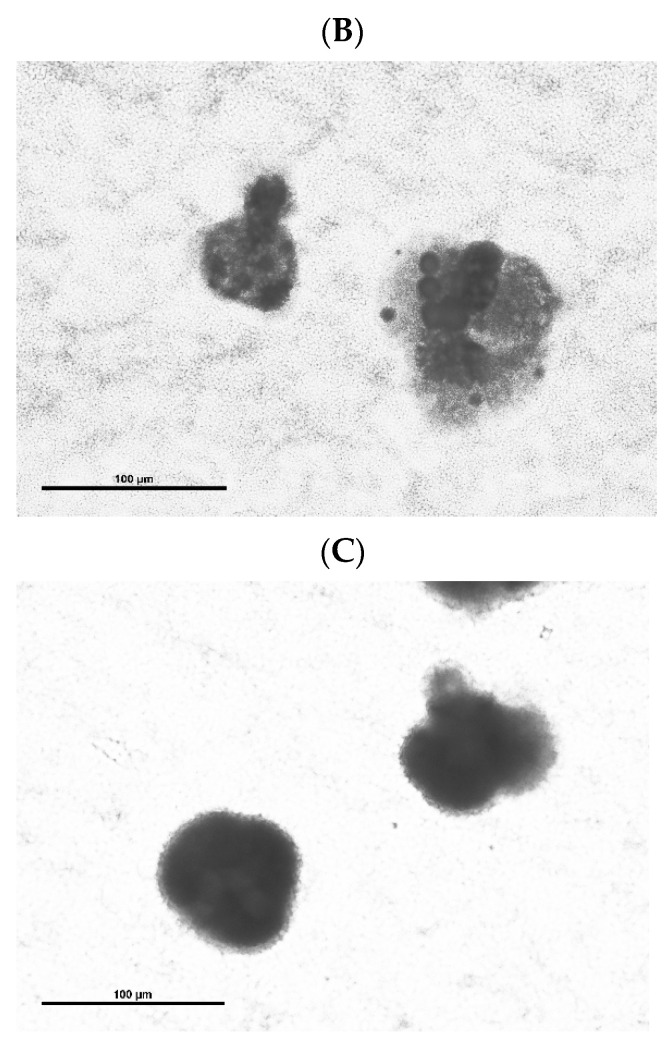
Picture taken under a microscope showing *M. tuberculosis* H37Rv (**A**) without peptide, (**B**) with peptide 14D at 1 µg/mL, and (**C**) with peptide 14D at 10 µg/mL.

We have found that two peptides were able, at 1 and 10 µg/mL, to diminish cord formation; see Table 2 and Figure 2.

## 4. Discussion

Tuberculosis (TB) remains a formidable global health challenge, with its persistent prevalence and the problem of drug-resistant strains. The current study delves into an innovative approach to TB management, focusing on the potential of antimicrobial peptides (AMPs) not just as antimicrobial agents but, more importantly, as growth stimulators for *Mycobacterium tuberculosis*. This unique approach aimed to reduce the Time to Positivity (TTP) in diagnostic tests, potentially leading to faster diagnosis and timely intervention.

The systematic substitution analysis of peptide 14D, which yielded 171 novel peptides, has illuminated a spectrum of interactions with *M. tuberculosis*. This provided a comprehensive insight into the sequence–activity relationship of these molecules. The extensive exploration has illuminated the nuanced interactions between these peptides and the bacterium. While some peptides further accelerated growth, others, intriguingly, inhibited it. This dual behaviour underscores the multifaceted nature of AMPs and their potential roles in both therapeutic and diagnostic applications. The peptide NH_2_-wkivfiwrr-CONH_2_, in particular, stands out for its ability to significantly reduce the Time to Positivity (TTP) by 25 h compared to the wild-type peptide. Such a reduction in TTP by just one amino acid substitution shows the potential to further improve peptides that can consequently improve TB diagnostics via culture. Faster detection and determination of the antibiogram translate to earlier interventions, which can be pivotal in improving patient outcomes and reducing transmission rates.

The data presents several noteworthy observations. Firstly, substitutions with alanine, glycine, proline, and, to a lesser extent, asparagine generally result in diminished growth-stimulating activity across most positions of the wild-type peptide. This trend appears to correlate with the peptide’s structural prerequisites for growth stimulation. The introduction of proline, which imparts significant rigidity and creates a hinge, tends to reduce activity. Similarly, the peptide’s growth-stimulating activity is compromised when its structure becomes overly flexible due to the incorporation of glycine or alanine.

Certain amino acids, when substituted, enhance the growth-stimulating activity across three or more positions. These include glutamic acid, isoleucine, lysine, and leucine. These amino acids are notably diverse: isoleucine and leucine are small and polar; glutamic acid is small and acidic; and lysine is larger and basic.

The presence of valine at position 4 appears pivotal for growth stimulation, as nine substitutions led to diminished activity, with no substitution enhancing the effect. Similarly, position 7 did not exhibit any activity augmentation, but unlike position 4, there was no marked decrease either. Substitutions at positions 1–3 and 7–9 did not result in any pronounced activity reduction, suggesting that the core sequence from positions 4–6 is paramount for interaction since only here were substitutions detected that flipped the growth stimulation effect into a strong inhibitory effect. However, position 5 appears suboptimal for the desired effect, as six substitutions led to heightened activity, the most significant increase observed across all peptide positions of the wild type.

**Figure 3:**
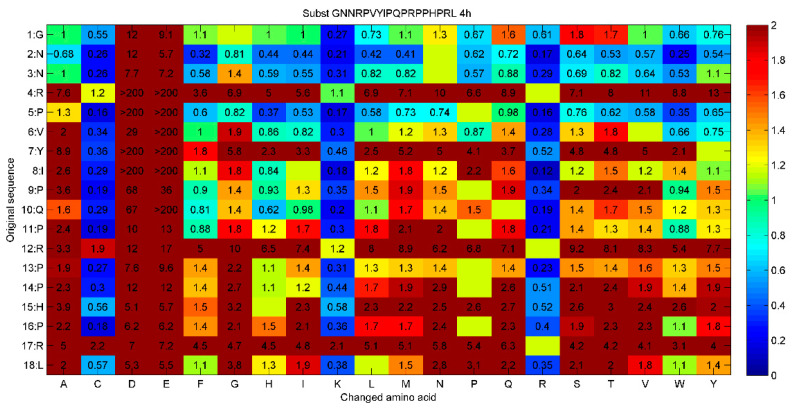
Analysis of amino acid substitutions in the antimicrobial peptide Apidaecin (GNNRPVYIPQPRPPHPRL). The wild type sequence and its respective positions are displayed in the first two columns. Subsequent rows (A–Y) show the changes in amino acids at each spot. The numbers inside each cell indicate the RelIC75 value, which is a measure of antimicrobial activity against *Pseudomonas aeruginosa* strain H1001 after four hours. The colour scheme of the cells ranges from blue to red: blue signifies enhanced activity compared to the original peptide, green denotes comparable activity, and red suggests no activity. Cells left blank represent the original sequence. This was taken from [65].

One of the most notable findings from the systematic substitution analysis of peptide 14D was the role of negatively charged amino acids in modulating *M. tuberculosis* growth. Historically, in substitution analyses performed on other AMPs targeting different pathogens, measuring antimicrobial activity, the introduction of negatively charged amino acids often resulted in a marked reduction in antimicrobial activity, see Figure 3. This is typically attributed to the disruption of electrostatic interactions between the positively charged AMPs and the negatively charged microbial cell membranes. However, in the context of measuring growth stimulation of *M. tuberculosis*, the opposite was observed. The incorporation of negatively charged amino acids, in many instances, enhanced the growth-stimulating activity of the peptides. This counterintuitive observation suggests that the interaction dynamics between these peptides and *M. tuberculosis* are distinct from those of conventional AMPs with their target pathogens. In addition, as seen in Figure 3, lysine and arginine both usually show improvements at many positions to increase antimicrobial activity. However, for the growth stimulation, except in one position, only lysine improves growth stimulation and contrary to expectations neither improves inhibition. These intriguing observations suggest a unique interaction mechanism between the peptide and the bacterium, which warrants further investigation.

Furthermore, the study identified peptides with strong inhibitory effects against *M. tuberculosis*, with a remarkable MIC of 20 µg/mL. This is particularly significant given the rise of multi-drug resistant strains of *M. tuberculosis*. The ability of these peptides to inhibit both the standard H37Rv strain and a multi-drug resistant strain at the same MIC underscores their potential as therapeutic agents. The consistent MIC against both strains suggests a mechanism of action that is not easily bypassed by the common resistance mechanisms present in the MDR strains. This could pave the way for the development of new therapeutic agents that can effectively combat resistant strains of *M. tuberculosis*. We believe that 20 µg/mL against a multi-drug resistant *M. tuberculosis* strain is a good starting point for further optimizations.

The observation of diminished cord formation in the presence of two peptides is another unexpected but significant finding. Cord formation is a well-documented virulence factor in *M. tuberculosis*, aiding in bacterial dissemination and potentially contributing to the spread of infection within the host. The ability of peptides to disrupt this process could have therapeutic implications, potentially limiting the spread of the bacterium within the host and making it more susceptible to immune clearance and antimicrobial therapy. It seems like the peptide 14D has an inherent ability to diminish cord formation and that other substitutions destroy this ability except for NH_2_-wkivf**d**wrr-CONH_2_. Further, more detailed investigations are necessary to understand this effect and its potential as a treatment option.

In conclusion, this study has provided valuable insights into the potential of peptide 14D and its variants in both the diagnostic and therapeutic realms of TB management. The findings underscore the versatility of AMPs and their potential to be harnessed in innovative ways to combat infectious diseases. Further studies are warranted to elucidate the exact mechanisms of interaction between these peptides and *M. tuberculosis*, which could pave the way for the development of novel diagnostic tools and therapeutic agents against this persistent global health threat.

## Figures and Tables

**Table 1:** Effect of purified peptide 14D and substituted variants at different concentrations on the time to positivity (TTP) of *M. tuberculosis* measured in MGIT in a BD BACTEC MGIT 320 system. All peptides are C-terminal amidated. For better comparison, peptides were presented in order according to their activity groups as shown in Figure 1. Substitutions with the potential for enhanced growth stimulation showed values less than 85% compared to the wild type; substitutions with the potential for medium growth inhibition showed values between 120% and 180% and substitutions with the potential for strong growth inhibition showed values exceeding 180% in the screen.

Sequence	TTP Values at Different Peptide Concentrations			
	0.3 µg/mL	3 µg/mL	10 µg/mL	30 µg/mL
TB alone				
12 d 4 h				
Wild-type peptide				
wkivfwwrr	10 d 17 h	10 d 21 h	11 d 21 h	12 d 04 h
Substitutions with the potential for enhanced growth stimulation				
**k**kivfwwrr	11 d 09 h	12 d 00 h	14 d 00 h	46 d 04 h
wkiv**d**wwrr	12 d 13 h	14 d 12 h	16 d 13 h	19 d 06 h
wkiv**k**wwrr	10 d 08 h	10 d 17 h	17 d 14 h	25 d 19 h
wkivf**i**wrr	10 d 16 h	9 d 20 h	10 d 07 h	12 d 21 h
wkivfwwr**e**	10 d 20 h	10 d 00 h	11 d 00 h	13 d 02 h
Substitutions with the potential for medium growth inhibition				
wki**a**fwwrr	11 d 01 h	12 d 19 h	20 d 16 h	47 d 06 h
Substitutions with the potential for strong growth inhibition				
wki**G**fwwrr	11 d 19 h	15 d 20 h	25 d 08 h	54 d 04 h
wki**s**fwwrr	10 d 00 h	14 d 08 h	22 d 11 h	53 d 20 h
wkivf**d**wrr	16 d 20 h	33 d 01 h	44 d 02 h	neg (55 d)
wkivf**G**wrr	12 d 17 h	22 d 08 h	46 d 06 h	neg (55 d)
wkivf**n**wrr	12 d 21 h	15 d 13 h	28 d 07 h	52 d 16 h

**Table 2:** Minimum inhibitory concentration (MIC) of peptides against *M. tuberculosis* H37Rv and a multi-drug resistant (MDR) strain determined via microdilution broth assay. Cord formation was observed with a Nikon Ni-E microscope fitted with a Nikon DS-Fi3 camera. The presence or absence of cord was checked at 10 µg/mL peptides and categorized. Cord formation like wild type (without peptide) was classified as “normal”. For two peptides, no cord formation was detected, and they were classified as “not observed”. All peptides are C-terminal amidated.

Sequence	MIC in µg/mL		Cord Formation at 10 µg/mL	
	H37Rv	MDR-TB	H37Rv	MDR-TB
wkivfwwrr	>50	>50	not observed	not observed
wki**s**fwwrr	20	20	normal	normal
wkivf**d**wrr	20	20	not observed	not observed
wkivf**G**wrr	20	20	normal	normal
wkivf**n**wrr	20	20	normal	normal

## Data Availability

The vast majority of data is presented in the manuscript. Further data presented in this study are available on request from the corresponding author.
